# Hyperlactatemia and other perioperative metabolic disturbances in neuroanesthesia

**DOI:** 10.1097/ACO.0000000000001180

**Published:** 2022-08-16

**Authors:** Markus Klimek, Peter de Smalen, Joost Janssen

**Affiliations:** Department of Anesthesiology, Erasmus University Medical Center, Rotterdam, The Netherlands

**Keywords:** acid–base-balance, cerebral salt wasting, lactate, neuroanesthesia, secretion of antidiuretic hormone

## Abstract

The concept of ‘brain-body cross-talking’ has gained growing interest in the last years. The understanding of the metabolic disturbances (e.g., hypernatraemia/hyponatraemia and hyperlactatemia) in neurosurgical patients has improved during the last years.

**Recent findings:**

The impact of elevated lactate without acidosis in neurosurgical patients remains controversial. The pathophysiology of inappropriate secretion of antidiuretic hormone (SIADH) has become clearer, whereas the diagnosis of cerebral salt wasting should be used more carefully.

**Summary:**

These findings will contribute to a better understanding of the pathophysiology involved and enable better prevention and therapy where possible in clinical practice.

## INTRODUCTION

Despite the presence of the blood-brain-barrier, there is a close interdependence between the brain and the composition of the circulating plasma. This is only one aspect of the so-called brain-body cross-talking, but an important one: on the one hand the aspects of the composition of the infused fluids, such as osmolarity, can interfere with the brain, whereas on the other hand, brain disorders such as diabetes insipidus and inappropriate secretion of antidiuretic hormone (SIADH) can cause severe disturbances in the plasma composition [1,2]. Finally, lactate has drawn special attention in neurosurgical patients. This article addresses the relevant metabolic disturbances in perioperative neuroanesthesia, the recent insights and their recommended current management. 

**Box 1:**
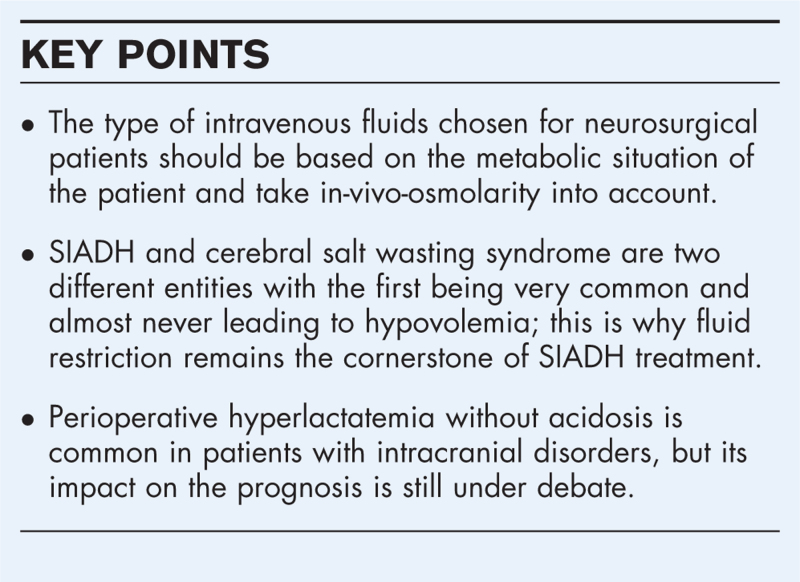
no caption available

## ACID–BASE BALANCE AND PERIOPERATIVE FLUID MANAGEMENT

According to the physicochemical approach to acid–base balance, as described by Peter Stewart, the metabolic component of acid–base balance is influenced by two independent factors: the strong ion difference (SID) and the total weak acid concentration (*A*_TOT_). The SID is calculated in simplified form by [Na^+^] + [K^+^] − [Cl^–^] − [lactate^–^]. The total weak acid concentration is mostly determined by albumin and phosphate concentrations. The human body provides an inexhaustible source of H^+^ through the partial dissociation of water, the extent of which is influenced by the SID and *A*_TOT_. A decrease in the SID, for example due to hyperchloremia or hyperlactatemia, will lead to an increase in the dissociation of water into H^+^ to maintain electroneutrality and will therefore lead to a decrease in pH [3].

As one of the strong ions, serum lactate has an important role in acid–base balance. The type B hyperlactatemia as witnessed in the neurosurgical population, could therefore result in decreased pH. However, the final effect on pH is always the result of the interplay between all components of the SID and *A*_TOT_. As shown in our recent study, normal pH does not mean normal acid–base balance as opposing disturbances in SID and *A*_TOT_ can balance each other out [4^▪^]. Furthermore, acid–base balance is a dynamic process. Postoperative metabolism of excess lactate to bicarbonate in neurosurgical patients will lead to an increase in SID and therefore potentially metabolic alkalosis. In fact, in the high-dependency unit of our hospital metabolic alkalosis was the most frequent acid–base disturbance on day one after surgery and occurred mostly in neurosurgical patients presenting with normal pH and concurrent type B hyperlactatemia.

Not only does the Stewart approach provide a framework for the analysis of acid–base disturbances, it also allows us to predict the effects of fluid therapy on acid–base balance through its influence on plasma SID and *A*_TOT_. Infusion of crystalloid solutions will lead to dilution of *A*_TOT_, and thus has an alkalizing effect, whereas the SID of the fluid infused will change plasma SID. This is the cause of the well-known hyperchloremic acidosis caused by 0.9% NaCl infusion. NaCl in any concentration is a fluid with a SID of zero, whereas plasma has a SID of approximately 40 mmol/l. To avoid infusion-fluid-induced acid–base disturbances the SID of the solution should be lower than the plasma SID to counteract the effect of the dilution of *A*_TOT_, ideally around 24 mmol/l, which is equal to the physiological bicarbonate concentration [5]. When the SID of the fluid infused is below 24 mmol/l a risk of acidosis is introduced.

Studies in adult and pediatric neurosurgical and non-neurosurgical patients have consistently shown that patients treated with 0.9% NaCl have a higher incidence of postoperative hyperchloremic acidosis compared with those treated with a balanced crystalloid solution. Kang *et al.* compared the use of balanced crystalloid versus 0.9% NaCl retrospectively in 586 patients undergoing clipping for unruptured intracranial aneurysm. The incidence of postoperative metabolic acidosis was significantly lower in those receiving primarily balanced crystalloids (0.7 versus 4.4%). Furthermore, the time until extubation in the intensive care unit was shorter (122 versus 250 min) [6]. Lima *et al.* performed a randomized controlled trial comparing balanced crystalloid to 0.9% NaCl in 52 children undergoing craniotomy for brain tumor resection. The use of 0.9% NaCl resulted in a higher incidence of hyperchloremia and subsequent hyperchloremic acidosis (24 versus 0%) [7].

Perioperative hyperchloremia has been associated with increased risk of acute kidney injury (AKI). Oh *et al.*[8] investigated this association retrospectively in 726 patients who underwent a craniotomy. They found hyperchloremia to be a risk factor for postoperative AKI. A second retrospective study from the same group in 968 patients who underwent craniotomy for intracranial hemorrhage only found hyperchloremia as a risk factor for postoperative AKI when it also resulted in acidosis (odds ratio 1.85; 95% confidence interval, 1.102–3.106) [9]. One should realize that both mannitol and hypertonic saline are infusion fluids with a SID of zero and therefore a potential contributor to acidosis, too.

From an acid–base perspective the ideal infusion fluid depends on the patient's current acid–base status. As a rule of thumb, when the SID of the fluid infused is lower than plasma bicarbonate, pH will decrease and vice versa. Additional factors to consider are the concurrent use of low SID fluids such as mannitol and hypertonic saline and the presence of hyperlactatemia. Of course, acid–base balance is not the sole concern in perioperative fluid management, one should also consider fluid osmolarity. This is especially true for the neurosurgical population in which brain edema due to hypotonicity should be avoided. As some of the balanced solutions are quite hypotonic *in vivo*, 0.9% NaCl has historically been the fluid of choice in neurosurgery. Table 1 provides an overview of the characteristics of crystalloid solutions to guide clinical decision making. A recent letter further emphasized the importance of these characteristics and advocated that the packaging of solutions should clearly state the physiological consequence of its use [10^▪^].

**Table 1:** Characteristics of plasma and common crystalloid solutions

	Plasma	NaCl 0.9%	Lactated Ringer's	Plasma-lyte 148	Sterofundin ISO	NaCl 3%	20% Mannitol
Na^+^ (mmol/l)	142	154	130	140	145	512	0
Cl^-^ (mmol/l)	103	154	109	98	127	0	0
K^+^ (mmol/l)	4.5	0	4	5	4	0	0
Ca^2+^ (mmol/l)	2.5	0	2.7	0	2.5	0	0
Mg^2+^ (mmol/l)	1.25	0	0	1.5	1	0	0
Buffer^a^ (mmol/l)	24	0	28	50	29	0	0
In-vivo SID (mmol/l)	40	0	27	50	25.5	0	0
In-vitro osmolarity (mOsm/L)	291	308	273	295	309	1026	1098
In vivo osmolality (mOsm/kg)	288	286	254	271	290	972	1378

SID, strong ion difference.

a Buffer in plasma is bicarbonate, in lactated Ringer's lactate, in plasma-lyte 148 acetate (27 mmol/l) and gluconate (23 mmol/l), in sterofundin ISO acetate (24 mmol/l), and maleate (5 mmol/l). Plasma-lyte 148 manufactured by Baxter Healthcare, Toongabie, NSW, Australia; Ringer's Lactate manufactured by Baxter Healthcare, Deerfield, IL, United States; Sterofundin ISO manufactured by B. Braun Melsungen AG, Melsungen, Germany.

## ELECTROLYTE AND FLUID DISTURBANCES IN PERIOPERATIVE NEUROANESTHESIA

The cerebral salt wasting syndrome (CSWS) has a long history of confusion and controversy regarding its very existence and prevalence [11^▪^]. Recently, several attempts have been made to get a clearer picture of CSWS. In any case, it is important to distinguish a disturbance of the sodium balance from a disturbance of the fluid balance. Today's most accepted view is that the natriuresis and hyponatremia that frequently accompany intracranial disease are due to an inadequate SIADH, which is a different disease-entity [12]. The vast majority of hyponatremia in patients with intracranial pathology is caused by SIADH, especially in the early phase of the disease. SIADH is typically seen in patients with subarachnoid hemorrhage treated with an over generous fluid-regimen (e.g., 3 l/day NaCl 0.9%), which at least partially triggers SIADH and can explain the attempt of the body to compensate for it. This mechanism also explains why hypovolemia is rare in patients with SIADH despite the natriuresis. Therefore, SIADH can easily be treated with fluid restriction which should nevertheless not lead to a severe hypovolemia due to depletion of the extracellular/intravascular fluid compartment.

Patients with CSWS always show intravascular hypovolemia. The reported incidence of CSWS in patients with intracranial pathology reflects its vague definition and varies between 0 and 94% [12]. Some authors describe an association between the incidence of CSWS and the severity of the intracranial pathology (assessed by computed tomography-scan or Glasgow Coma Score (GCS)), but this is debated [13]. Although the pathophysiology of SIADH is clearly defined by its name, the involved mechanisms in CSWS are less clear. At least five natriuretic peptides have been identified to be associated with CSWS: atrial natriuretic peptide, brain natriuretic peptide, C-type natriuretic peptide, dendroaspis natriuretic peptide, and ouabainlike peptide [13]. Furthermore, a reduction of the sympathetic input to the kidneys seems to play another important role in CSWS. Both mechanisms finally increase the glomerular filtration rate, inhibit the sodium reabsorption in the renal tubules and suppress the renin–angiotensin–aldosterone system. The latter further inhibits sodium retention and can be present without intracranial pathology. In that case the term ‘renal salt wasting syndrome’ (RSWS) is used [13].

It should be clear that both entities, SIADH and CSWS, lead to a hypoosmolar hyponatremia. Therefore, measuring just serum and urine sodium and osmolarity will not be sufficient. However, while SIADH leads to eu- or hypervolemia, CSWS will always cause hypovolemia. Assessing the fraction of urate excretion can also be helpful. CSWS or RSWS are characterized by an increased urate excretion, whereas it is usually normal in the case of SIADH [14]. However, this is debated, and some authors use the response of the patient on isotonic saline to distinguish between SIADH (urine becomes concentrated) and CSWS/RSWS (urine becomes diluted). The same authors also recommend abandoning the term CSWS and to use RSWS for the combination of natriuresis with hypovolemia [11^▪^].

**FIGURE 1:**
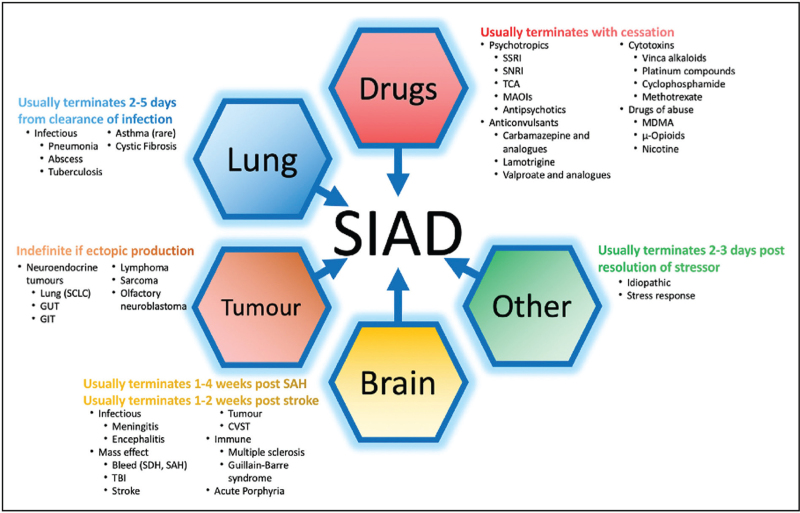
Common causes of the syndrome of inappropriate antidiuresis. Typical duration of syndrome of inappropriate antidiuresis caused by each cause is shown in gray. AVP, arginine vasopressin (antidiuretic hormone); CVST, cerebral venous sinus thrombosis; GIT, gastrointestinal tract; GUT, genitourinary tract; MAOIs, monoamine oxidase inhibitors; MDMA, 3,4-methylenedioxy-methamphetamine; SAH, subarachnoid hemorrhage; SCLC, small cell lung carcinoma; SDH, subdural hematoma; SIAD, syndrome of inappropriate antidiuresis; SNRI, serotonin noradrenaline reuptake inhibitor; SSRI, selective serotonin reuptake inhibitor; TBI, traumatic brain injury; TCA, tricyclic antidepressant. Reproduced with permission [16^▪▪^].

Based on the pathophysiological mechanisms described above, the therapeutic approaches are completely different: patients with CSWS are treated with isotonic saline, whereas loop diuretics or fluid restriction will help patients with SIADH. Tolvaptan, a vasopressine-V2-receptor-antagonist, was introduced more than 10 years ago, but is still more experimental than a routine drug in SIADH-treatment. Nonetheless, tolvaptan has recently shown beneficial effects in patients with SIADH after subarachnoid hemorrhage [15]. Current guidelines recommend tolvaptan as an escape-medication in resistant hyponatremia without hypovolemia [16^▪▪^]. It must be stressed that SIADH has many causes other than intracranial pathology, as Fig. 1 summarizes [16^▪▪^].

## DIABETES INSIPIDUS

Only a few new insights relevant to neuroanesthesiological practice will be presented here:

First, the diagnosis of diabetes insipidus, should be considered carefully and more frequently, especially in children, as it can be a relevant comorbidity in pediatric patients [17]. Second, the measurement of the serum-marker copeptin has been shown to be more reliable than arginin-vasopressin in patients with hypernatraemia. This might be of added value in the postoperative evaluation of polyuric patients after intracranial surgery [18^▪^].

## LACTATE

As mentioned above, lactate is an important anion with physiology relevant to neuroanesthesia. In normal cell metabolism, lactate has a role in the production of ATP; the fuel of life. Within the cell, glucose is metabolized to pyruvate by glycolysis. In the presence of oxygen, pyruvate will be metabolized in the Krebs cycle via acetyl-coenzyme A (oxidative phosphorylation). With this process ATP is generated. Pyruvate, however, can also be metabolized to lactate by the enzyme lactate dehydrogenase (LDH). Normally, this reaction is balanced and the lactate–pyruvate ratio is regulated. This process also generates ATP, albeit much less efficiently than oxidative phosphorylation. In the absence of oxygen, the anaerobic metabolism will lead to a shift of the lactate–pyruvate ratio and lactate will accumulate, lowering the pH. In the short term this can provide the cell with ATP, but in the longer term the anaerobic metabolism leads to apoptosis. This is referred to as hyperlactatemia type A. In contrast, hyperlactatemia type B is defined as hyperlactatemia unrelated to tissue hypoxia. In pathophysiological conditions (e.g., when intracranial tumors are present), the above mechanism changes. Already in the last century a form of aerobic glycolysis was described, which was later termed the Warburg effect, named after its discoverer. Even in the presence of oxygen, malignant cells use the fermentation of pyruvate to lactate for their energy supply. This mechanism stimulates angiogenesis, growth, division, and survival of the cell.

Preoperatively, in patients with an intracranial process, lactate can be used to better estimate the nature of the tumor. For example, in patients with a high-grade tumor and in patients with cerebral metastasis, serum lactate appears to be elevated [19–22]. The increased lactate production and acidic microenvironment are also used in radiological diagnostics. Tissue lactate can be detected by magnetic resonance spectroscopy. Recently, steps have been taken to make these diagnostic processes more reliable [23]. Detection of cerebral lactate with magnetic resonance spectroscopy has been repeatedly associated with poorer survival [24]. One might logically think that elevated serum lactate would also be associated with poorer survival, but these results are contradictory [22,25].

Discovery of the Warburg effect has also led to innovative treatments. For example, the phosphoinositide 3-kinase (PI3K) inhibitors may limit tumor growth by acting on the PI3K-AKT pathway. Activation of this pathway could make the tumor more sensitive to glucose depletion [26]. Another pathway of special interest is that of the acid-sensing ion channel 1a. This channel may play an important role as a tumor suppressor in gliomas because it responds to an acidic extracellular environment [27].

Intraoperative hyperlactatemia is associated with preoperative elevated serum lactate as a manifestation of tumor type. In our 2020 study, we reported a mitigating effect of propofol on serum lactate levels [28]. This is probably due to the direct effect of propofol on LDH. However, influence of choice of anesthetic type – total intravenous anesthesia versus inhalational anesthesia – on the development of hyperlactatemia has only been studied to a limited extent. Most studies agree that intraoperative hyperlactatemia is not associated with neurological decline or worse patient survival [29]. However, there are consistent indications that patients with hyperlactatemia have a longer hospital stay than patients with a normal intraoperative serum lactate [30,31]. A well described cause for the longer hospital admission is lacking. However, it is evident that a longer admission is more demanding for healthcare staff and ultimately costs more.

Like intraoperative hyperlactatemia, postoperative hyperlactatemia was associated with a longer hospital stay, but not with an increase in neurological deterioration [28,32]. The exact cause of this has not been investigated. The question remains whether patients with postoperative hyperlactatemia have a greater burden of disease than the patients without hyperlactatemia, who leave the hospital earlier. Another possibility is that more diagnostic tests are performed and/or treatments are used because of the ‘abnormal’ serum lactate, without a clear clinical necessity.

As expected, postoperative hyperlactatemia is also related to tumor type. Malignant glioblastomas have been shown to lead to a higher serum lactate level [20,28,32]. Several studies also report that postoperative hyperlactatemia is aggravated by steroid use, duration of surgery and blood glucose levels [28,32,33]. Both due to the action of corticosteroids and an increase in blood glucose levels, there will be an increased supply of pyruvate. Automatically, more lactate is produced, but the pyruvate–lactate ratio remains within the normal range. Three studies reported that the surgery duration was related to postoperative hyperlactatemia on univariable analysis. After adjusting for confounders, only two studies showed a significant correlation for surgery duration and postoperative hyperlactatemia [28,33]. The influence of surgery duration remains debatable. This is further fueled by another study that reports no significant impact of surgery duration on intraoperative hyperlactatemia [31].

Technological advancement has made it possible to measure lactate noninvasively [34]. Saliva and sweat are among the most commonly researched bodily fluids. Before clinical application is possible and feasible, however, some hurdles must be overcome. For example, it is not clear whether the lactate in fluids corresponds to the serum lactate and what the kinetics are. Nonetheless, the ability to measure lactate virtually continuously will provide us with a wealth of information and this will hopefully lead to better understanding and clinical management.

In the physiological resting situation lactate provides about 10% of the brain's metabolism. In healthy volunteers this increases to about 25% during exercise. In patients who are comatose following traumatic brain injury, approximately 57% of the energy required is obtained from lactate [35]. With this in mind, studies have been conducted where lactate was intravenously administered in patients with traumatic brain injury or sub-arachnoid hemorrhage. Infusion of lactate increases brain-derived neurotrophic factor (BDNF). BDNF is neuroprotective; it facilitates neurogenesis, neuroregeneration, synaptic plasticity and memory. In addition, lactate binds to receptor GPR81 on the surface of the cell, thereby blocking activation of inflammatory pathways.

In conclusion, for brain tumor surgery, hyperlactatemia has not been associated with worse outcomes. However, lactate has been consistently associated with prolonged hospital stay. We would advise against aggressive treatment of hyperlactatemia type B. In fact, in the future we might artificially elevate serum lactate, to provide neuroprotection.

## CONCLUSION

The current overview addressed several recent and relevant aspects of metabolic disturbances that can be found in neurosurgical patients. Some of them are preventable, not all are easily treatable. Understanding the pathophysiology involved will help to improve the perioperative management.

## Acknowledgements

*None.*

### Financial support and sponsorship

*The current work was supported by the Department of Anesthesiology, Erasmus University Medical Center, Rotterdam, The Netherlands.*

### Conflicts of interest

*There are no conflicts of interest.*

## REFERENCES AND RECOMMENDED READING

Papers of particular interest, published within the annual period of review, have been highlighted as:

▪ of special interest
▪▪ of outstanding interest
